# Identification and Characterization of Dpo42, a Novel Depolymerase Derived from the *Escherichia coli* Phage vB_EcoM_ECOO78

**DOI:** 10.3389/fmicb.2017.01460

**Published:** 2017-08-02

**Authors:** Zhimin Guo, Jing Huang, Guangmou Yan, Liancheng Lei, Shuang Wang, Ling Yu, Liang Zhou, Anchong Gao, Xin Feng, Wenyu Han, Jingmin Gu, Junling Yang

**Affiliations:** ^1^Department of Respiratory Medicine, The Second Hospital of Jilin University Changchun, China; ^2^Department of Clinical Laboratory, The First Hospital of Jilin University Changchun, China; ^3^Key Laboratory of Zoonosis Research, Ministry of Education, College of Veterinary Medicine, Jilin University Changchun, China; ^4^Agricultural Experiment Base, Jilin University Changchun, China

**Keywords:** bacteriophage, depolymerase, *Escherichia coli*, biofilm, beta-lactamase

## Abstract

Biofilm formation, one of the most important virulence factors of pathogenic bacteria, protects bacteria against desiccation, antibiotics, phages and host immune responses. However, phage-derived depolymerases show antibiofilm activity and demonstrate great potential to treat infections caused by biofilm-forming bacteria. In this study, the *Escherichia coli* phage vB_EcoM_ECOO78 was isolated and characterised, and we observed its ability to lyse five out of 34 tested *E. coli* clinical isolates. The highest phage titre was observed at a multiplicity of infection of 10^-5^ and a burst size of approximately 74 plaque forming units (PFU)/infection. Electron micrographs indicated that vB_EcoM_ECOO78 belongs to the family *Myoviridae*. The presence of increasing halos surrounding the lysis plaques formed by vB_EcoM_ECOO78 indicated that this phage may encode a depolymerase. Based on a sequencing analysis, the complete genome of vB_EcoM_ECOO78 was found to be 41,289 bp in size, with a GC content of 53.07%. Additionally, vB_EcoM_ECOO78 has 56 predicted open reading frames, 51 (91.07%) of which are assumed to be functional. A BLAST analysis indicated that ORF42 of vB_EcoM_ECOO78 (Dpo42) has low identity with other reported phage-associated depolymerases. Dpo42 was expressed and purified as a soluble protein using *E. coli* BL21. The biofilm formation ability of *E. coli* isolates and the antibiofilm activity of Dpo42 were tested by performing spot assays and using a 96-well micro-titre plate method. Dpo42 degraded the capsular polysaccharides surrounding *E. coli* and exhibited dose-dependent biofilm-formation prevention activity. Based on these results, Dpo42 appears to be a novel phage-derived depolymerase that represents a new potential strategy for preventing *E. coli* biofilm formation.

## Introduction

Extended-spectrum beta-lactamase (ESBL)-producing *Escherichia coli* is a common Gram-negative pathogen in China (Zhang et al., 2016) that causes community- and hospital-acquired infections. The number of ESBL-positive *E. coli* strains has significantly increased in recent years (Canton et al., 2008). One of the most important virulence factors of *E. coli* is the ability to form biofilms. There are three necessary steps involved in biofilm formation: initial adhesion, early development of biofilm and maturation of the developed biofilm (Sharma et al., 2016). The structure and composition of bacterial biofilms were first described in Zobell (1943), and vary between species or strains based on biofilm age and environmental conditions (Harper et al., 2014). Usually, biofilms are microbial communities attached to a surface and encased in an extracellular matrix mainly composed by extracellular polymeric substances (EPSs), but also by proteins, nucleic acids, lipids, water and mineral ions (Costerton, 1995; O’Toole et al., 2000; Flemming and Wingender, 2010). The EPSs can be capsular polysaccharides or biofilm matrix polysaccharides (Latka et al., 2017). Three major EPSs have been reported, including β-1,6-*N*-acetyl-D-glucosamine polymer (PGA), cellulose and colanic acid, that comprise the *E. coli* biofilm matrix (Sharma et al., 2016). For *E. coli*, more than 80 distinct serotypes have been established depending on their capsular polysaccharides (Whitfield and Roberts, 1999). The ability of bacteria to form biofilms represents a serious medical challenge given the increasing prevalence of ESBL-positive *E. coli* strains. Therefore, there is a need to identify and develop new therapeutic strategies to eradicate *E. coli* biofilms (Stewart and Costerton, 2001).

Phages are viruses that can specifically infect bacteria. Therefore, in recent years, lytic phages have been reconsidered for the treatment of multidrug-resistant bacteria infections and several clinical trials have proved a great potential exists (Summers, 2001; Chanishvili, 2012; Oliveira et al., 2015). Further more, phage-derived proteins (such as lysins and holins) have been developed as antibacterial agents *in vitro* and *in vivo* (Nelson et al., 2001; Fischetti, 2005). Although numerous studies have investigated phages and lysins, little is known about phage depolymerases. Phage depolymerases are enzymes encoded by phages that specifically degrade bacterial EPSs (Latka et al., 2017). The existence of depolymerase is commonly identified by the development of an increasing halo surrounding each lysis plaque after prolonged incubation (Cornelissen et al., 2012; Harper et al., 2014; Pires et al., 2016). Additionally, phage-derived depolymerases potentially control the formation of *E. coli* biofilms (Stewart et al., 1995). A recent review reported on the putative depolymerases present in phages infecting different bacterial genera (Pires et al., 2016); among these phages, only a few *E. coli* phages produced depolymerases. In fact, we have discovered that more and more putative depolymerases can be found.

The activities of depolymerases extracted from phage lysates against serotype K30 capsular *E. coli* polysaccharide have been described (McCallum et al., 1989). The bacteriophage K1F tail protein is a depolymerase (endo-*N*-acylneuraminidase) that degrades the α-(2, 8)-linked polysialic acid chains of *E. coli* K1 serotype (Petter and Vimr, 1993). In 2004, bacteriophage-derived endosialidase E (endoE) was expressed and purified, and was observed to selectively degrade the polysialic acid capsule on the surface of *E. coli* K1 strains (Mushtaq et al., 2004). Besides these, other bacterial phage-derived depolymerases have also been studied, including *Enterobacter agglomerans* phage depolymerase (Hughes et al., 1998a), *Erwinia amylovora* phage depolymerase (DpoL1) (Born et al., 2014), *Acinetobacter baumannii* phage depolymerase ORF40ΦAB6 (Lai et al., 2016), *Staphylococcus epidermidis* phage depolymerase Dpo7 (Gutierrez et al., 2015) and *Klebsiella* phage depolymerase depoKP36 (Majkowska-Skrobek et al., 2016).

In this study, an *E. coli* phage was isolated and a novel phage-derived depolymerase, Dpo42, was characterised. Dpo42 could degrade the capsular polysaccharides surrounding *E. coli* and exhibited biofilm-formation prevention activity *in vitro*.

## Materials and Methods

### Bacterial Strains and Drug Resistance

The *E. coli* ATCC 25922 strain was purchased from the American Type Culture Collection (ATCC). Drug-resistant *E. coli* isolates were isolated from patients at the First Hospital of Jilin University (Changchun, Jilin province, China). The antibiotic susceptibility of the *E. coli* isolates was evaluated using the VITEK^®^ 2 Compact system (bioMérieux, Marcy-l’Étoile, France). Glycerol bacterial stocks were stored at -80°C and transferred to lysogeny broth (LB) medium (BioCorp, Warszawa, Poland) before recovery at 37°C. Tryptic Soy Broth (TSB) medium (bioMérieux, Marcy-l’Étoile, France) was used to culture bacterial biofilms. All strains were tested to determine the host range of the phage and to obtain the depolymerase antibiofilm activity.

### Isolation and Purification of Phage Particles

Sewage samples were obtained from the First Hospital of Jilin University in Changchun. Phage isolation and purification were performed as described previously, with minor modifications (Lin et al., 2010). Each 100-mL water sample was filtered using a sterile 0.22 μm Filter (Millex-GP Filter Unit; Millipore, Bedford, MA, United States; LOT R6MA05262) to remove any impurities and microorganisms. Then, the filtered solution was utilised to prepare LB liquid medium (tryptone 1%, yeast extract 0.5%, NaCl 1%) instead of purified water. Host *E. coli* O78-3 cells were cultured to the logarithmic phase, added to LB liquid medium and mixed. The culture was centrifuged at 4°C and 3,762 × *g* for 15 min after incubation at 37°C for 18 h. The supernatant was then filtered using a sterile 0.22 μm philtre. Phages were purified using the double-layer agar plate method. A total of 100 μL of overnight-cultured *E. coli* O78-3 and phage were added to melted semi-solid medium (tryptone 1%, yeast extract 0.5%, NaCl 1%, agar 0.7%) (45°C) and immediately mixed, then poured onto plates containing LB solid medium (tryptone 1%, yeast extract 0.5%, NaCl 1%, agar 1.5%). The plates were cultured at 37°C for 12 h. Phages were purified until the plaques were uniform in size. Phage lysates were centrifuged at 4°C at 3,762 × *g* for 30 min. The purified phages were stored in Suspended Medium (SM) buffer (gelatine 0.01%, NaCl 100 mmol/L, Tris-HCl 50 mmol/L, and MgSO_4_ 10 mmol/L) at 4°C. A total of 1 μg/mL RNase A and DNase I (Sigma–Aldrich, St. Louis, MO, United States) was added into the phage supernatant, and then the mixture was incubated for 30 min at room temperature. To separate bacterial fragments and phage particles, 1 mol/L NaCl was added to the supernatant on ice and incubated for 1 h. The supernatant was then centrifuged at 4°C and 10,451 × *g* for 10 min. To precipitate the phage particles, 10% (w/v) PEG 8000 was added to the supernatant and gently mixed on ice, followed by incubation for 3 h. Samples were centrifuged at 10,451 × *g* for 10 min, resuspended in SM buffer, extracted with chloroform to remove bacterial debris, and centrifuged at 4°C and 3,762 × *g* for 10 min.

### Transmission Electron Microscopy

Phages were concentrated via CsCl density gradient (1.32, 1.45, 1.5, and 1.7 g/mL) centrifugation at 4°C and 35,000 × *g* for 3 h (Russell, 2001). The purified phages were dropped onto a 200-mesh copper film on a slide and negatively stained with phosphotungstic acid (2% w/v). A transmission electron microscope (JEOL JEM-1200EXII; Japan Electronics and Optics Laboratory, Tokyo, Japan) was used to observe the samples at an acceleration voltage of 80 kV.

### Host Spectrum Determination

The host range of vB_EcoM_ECOO78 was determined by performing the spot test and double-layer agar plate test (Barrow et al., 1998). For the double-layer agar plate test, dilutions of phages and the tested *E. coli* strains were added to melted semi-solid medium (45°C) and immediately mixed, then poured onto LB solid medium plates. To perform the spot test, 200 μL of overnight-cultured *E. coli* were coated on LB solid medium plates and allowed to dry. Then, 10 μL of phage suspension was dropped onto each plate, and the plates were cultured at 37°C for an additional 10 h to observe the formation of clear spots.

### Determination of Multiplicity of Infection (MOI)

The MOI was detected as described by Gu et al. (2011). Briefly, *E. coli* O78-3 host cells were cultured to logarithmic phase, and the phage was inoculated into liquid LB medium containing the host bacteria at MOIs of 10, 1, 10^-1^, 10^-2^, 10^-3^, 10^-4^, 10^-5^, or 10^-6^. The bacteria and phage mixtures were cultured at 37°C for 12 h. Phage titres in the filtrates were measured. The experiment was repeated three times.

### One-Step Growth Curve Assay

A one-step growth curve was constructed as described previously by Gu et al. (2011), with minor modifications. *E. coli* O78-3 host cells were grown to the mid-logarithmic phase. Phages were added at an MOI of 10^-5^, followed by incubation for 15 min at 4°C. The mixture was centrifuged at 4°C and 15,049 × *g* for 30 s, and the pellet was suspended in 10 mL of fresh LB liquid medium. The samples were then incubated at 37°C at 180 rpm. Samples were taken every 5 min for the first 30 min, and at 40, 60, 80, 90, 100, 120, and 150 min to test phage titration. Tests were conducted three times. The burst size was defined as the ratio of the mean number of phage in final rise phase to the mean number of phage in the latent period.

### DNA Sequencing and Analyses

The method used to extract the vB_EcoM_ECOO78 genome has been reported previously (Russell, 2001). Briefly, the concentrated phage was treated with DNase I (final concentration, 10 μg/mL) and RNase A (final concentration, 5 μg/mL) in SM buffer at 37°C for 1 h. Subsequently, ethylenediaminetetraacetic acid (EDTA) was added (pH 8.0, final concentration 25 mmol/mL). Finally, the phage genomic DNA was extracted using a Viral DNA Kit (Omega Bio-Tek^®^).

Whole-genome sequencing was performed by the Suzhou GENEWIZ Biotechnology Co., Ltd. using an Illumina HiSeq 2500 sequencer and SOAPdenovo v2.01 stitching to obtain the complete genome sequence of vB_EcoM_ECOO78. The identification of coding regions in the vB_EcoM_ECOO78 genome was based on the application of ORF Finder^1^. Open reading frames (ORFs) were automatically predicted using Glimmer^2^ and Genemark (Besemer and Borodovsky, 1999). The genome map of vB_EcoM_ECOO78 was drawn using CLC Main Workbench version 7.7.3 (CLC Bio-Qiagen, Aarhus, Denmark). To improve genome annotations/predictions, all proteins encoded by ORFs in the genome sequence of vB_EcoM_ECOO78 were subjected to a BLASTP search against the phage protein database at the National Center for Biotechnology Information^3^ and Pfam^4^. PSI-BLAST^5^ was used to predict the genes encoding the depolymerase. Phyre2^6^ was used to predict the structure of the depolymerase(Kelley et al., 2015).

### Expression and Purification of the Phage Depolymerase

ORF42, a putative phage depolymerase, was amplified from the purified phage vB_EcoM_ECOO78 by PCR using the primers Dpo42PF (5′-CGCGGATCCGTGGAACTGAAAACAT-3′) and Dpo42PR (5′-CCGCTCGAGTTAAATCAAGCCATAAATAGC-3′). Underlined nucleotides indicate the recognition sequences for BamHI and XhoI. The PCR fragment was excised with BamHI and XhoI and inserted into the pET-28a expression vector (Novagen, Madison, WI, United States). The constructed plasmid was used to transform *E. coli* BL21 cells. The ORF42 protein was expressed under 0.1 mM isopropyl β-D-1-thiogalactopyranoside (IPTG) induction at 16°C overnight, purified from the soluble fraction using a Ni-nitrilotriacetic acid (NTA) column (Genscript, Nanjing, China) according to the manufacturer’s instructions, and then analyzed using sodium dodecyl sulphate polyacrylamide gel electrophoresis (SDS-PAGE) (Gu et al., 2014). Lysis buffer was composed of 50 mM Na_2_HPO_4_, 0.3 M NaCl, pH 8.0; the wash buffer was composed of 50 mM Na_2_HPO_4_, 0.3 M NaCl, pH 8.0 with 50 mM imidazole; the elution buffer was composed of 50 mM Na_2_HPO_4_, 0.3 M NaCl, pH 8.0 with 250 mM imidazole. Subsequently, the purified Dpo42 was diluted in sterile phosphate-buffered saline (PBS; 137 mM NaCl, 2.7 mM KCl, 10 mM Na_2_HPO_4_, 1.8 mM KH_2_PO_4_, pH 7.2).

### Biofilm Assay

The biofilm formation of *E. coli* isolates was evaluated using the 96-well micro-titre plate method as described previously (O’Toole et al., 1999), with some modifications. Briefly, *E. coli* isolates were inoculated into 3 mL of sterile TSB medium and grown for 16 h at 37°C. A 1:100 dilution of each *E. coli* culture was created in sterile TSB medium, and 200 μL of each diluted culture was added to six wells in untreated 96-well micro-titre plates (Nest; NEST Biotech Co, China). A total of 200 μL of fresh TSB medium was used as a negative control. The plates were incubated at 37°C for 72 h without agitation. Non-adherent cells were removed by pipetting out the culture and washing the wells twice with 200 μL of sterile PBS. Then, 200 μL of methanol was added to each well, and the plates were incubated for 30 min at room temperature. The methanol was removed, and the plates were dried at room temperature. Subsequently, 200 μL of 1% w/v crystal violet solution was added to each well, and the plates were incubated for 30 min at room temperature. Wells containing the biofilm total biomass were washed gently with sterile deionized water. The plates were left to dry at room temperature. Thereafter, 200 μL of 33% v/v glacial acetic acid was added to each well to solubilize the bound crystal violet from the stained *E. coli* biofilms. The optical density (OD) was measured at 590 nm using a Synergy 2 multi-mode microplate reader (BioTek, United States). The isolates were classified as having strong (4 × ODc ≤ OD), moderate (2 × ODc < OD ≤ 4 × ODc), weak (ODc < OD ≤ 2 × ODc) or non-biofilm formation (OD ≤ ODc) based on a previous report (Stepanovic et al., 2000). ODc is a cut-off of the OD measurement obtained in the negative control three times. The experiments were performed in triplicate, and the results are expressed as the means ± SDs.

### Activity of Dpo42 against Biofilms

A spot test was performed to observe Dpo42 activity as described previously (Verma et al., 2009). In brief, LB agar was overlaid with 0.7% top agar inoculated with 200 μL of fresh log-phase bacterial culture. After drying, the purified Dpo42 (1 μg) was spotted onto the double-layer agar plate. Serial dilutions of Dpo42 (50, 5, 2.5, 1.25, 0.625, 0.3125, 0.156, and 0.078 ng) were also dropped onto host strain double-layer agar plates. The elution buffer diluted in PBS was used as a control. After overnight incubation at 37°C, plates were observed for the formation of semi-clear spots.

The lytic activity of Dpo42 (0.25 μg/μL) toward *E. coli* isolates was tested at 37°C (Gu et al., 2014). Cell counts were performed at 0 min, 30 min, 1, 2, and 4 h. The elution buffer diluted in PBS was used as a negative control.

*Escherichia coli* isolates E9 and HXM were used to test the preventative effects of Dpo42 on biofilm formation as described previously (O’Toole et al., 1999), with some modifications. Briefly, *E. coli* E9 and HXM were cultured to logarithmic phase, centrifuged, resuspended in equal amounts of TSB medium, mixed and (100 μL) added to each well of a 96-well micro-titre plate. The wells were then divided into four groups. The elution buffer diluted in PBS, 10 μg/well Dpo42, 25 μg/well Dpo42 or 50 μg/well Dpo42 at the volume of 100 μL was added to different groups; 200 μL of sterile TSB medium was used as a negative control. Each group contained triplicate samples. The plate was covered, and bacteria were permitted to adhere and grow at 37°C for 24, 72, and 96 h without agitation. After incubation, the residual biofilms were stained, and the OD_590_ was measured.

The effects of Dpo42 on 24 and 96 h old biofilms were also detected. *E. coli* E9 and HXM were cultured in the 96-well micro-titre plate to form 24 h old biofilm and 96 h old biofilm. The biofilms were treated with 10 μg/well Dpo42, 25 μg/well Dpo42, 50 μg/well Dpo42 or elution buffer diluted in PBS for 12 h. As a control, 200 μL of sterile TSB medium was used. Each group contained triplicate samples. After incubation, the residual biofilms were stained, and the OD_590_ was measured.

### Capsule Staining

The capsule staining of *E. coli* E9 was negatively stained with Maneval’s solution and analyzed by microscopy (Cornelissen et al., 2011). Cells grown in the absence and presence of Dpo42 were transferred to 10 μL of 1% aqueous Congo red solution (Sigma–Aldrich, St. Louis, MO, United States) and mixed. The mixture was spread across a glass slide to form a thin film and then air dried. Another 10 μL of Maneval’s solution (3.33% phenol, 4.44% glacial acetic acid, 2.67% ferric chloride, 0.02% acid fuchsin; Sigma–Aldrich) was dropped across the glass slide. Capsules were negatively stained and appeared white underneath the microscope (100×, oil; Olympus CX-41; Olympus America, Center Valley, PA 18034-0610, United States).

### Phylogenetic Tree Analysis

The terminase large subunit is one of the conserved phage proteins (Feiss and Rao, 2012). Thus, a phylogenetic tree analysis of vB_EcoM_ECOO78 was performed based on terminase large subunit sequences. The evolutionary history of the taxa was drawn using the neighbour-joining (NJ) method (Saitou and Nei, 1987) to construct a bootstrap consensus tree from 1,000 replicates. Evolutionary distances were computed using the Poisson correction method (Zuckerkandl and Pauling, 1965) and are indicated as the units for the number of amino acid substitutions per site. Evolutionary analyses were carried out using MEGA7 (Kumar et al., 2016).

Phylogenetic relationships were determined based on the protein sequences for Dpo42 and 10 other phage depolymerases. A molecular phylogenetic analysis was performed using the maximum-likelihood method. Additionally, the evolutionary history was inferred using the maximum-likelihood method based on the Poisson correction model (Zuckerkandl and Pauling, 1965). The tree with the highest log likelihood (-7059.4930) is shown. Initial tree(s) for the heuristic search were obtained automatically by applying the NJ and BioNJ algorithms to a matrix of pairwise distances estimated using a Jones–Taylor–Thornton (JTT) model, then selecting the topology with superior log likelihood values. Evolutionary analyses were carried out using MEGA7 (Kumar et al., 2016).

### Statistical Analysis

One-way analysis of variance (ANOVA) was performed for the normally distributed data. The qualitative data were analyzed using the chi-square and two-sided Fisher’s exact tests. *P*-values < 0.05 were considered statistically significant. All tests were performed using SPSS version 19.0 (SPSS 111 Inc., Chicago, IL, United States).

## Results

### General Microbiological Characteristics of vB_EcoM_ECOO78

**FIGURE 1:**
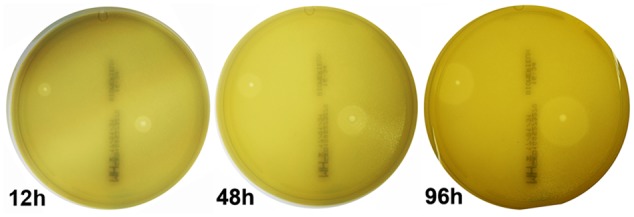
The appearance of plaques formed by phage vB_EcoM_ECOO78 on *E. coli* O78-3 biofilms. Pure phage plaques appeared after 12 h, and the haloes surrounding the phage plaques grew in size after 48 and 96 h.

**FIGURE 2:**
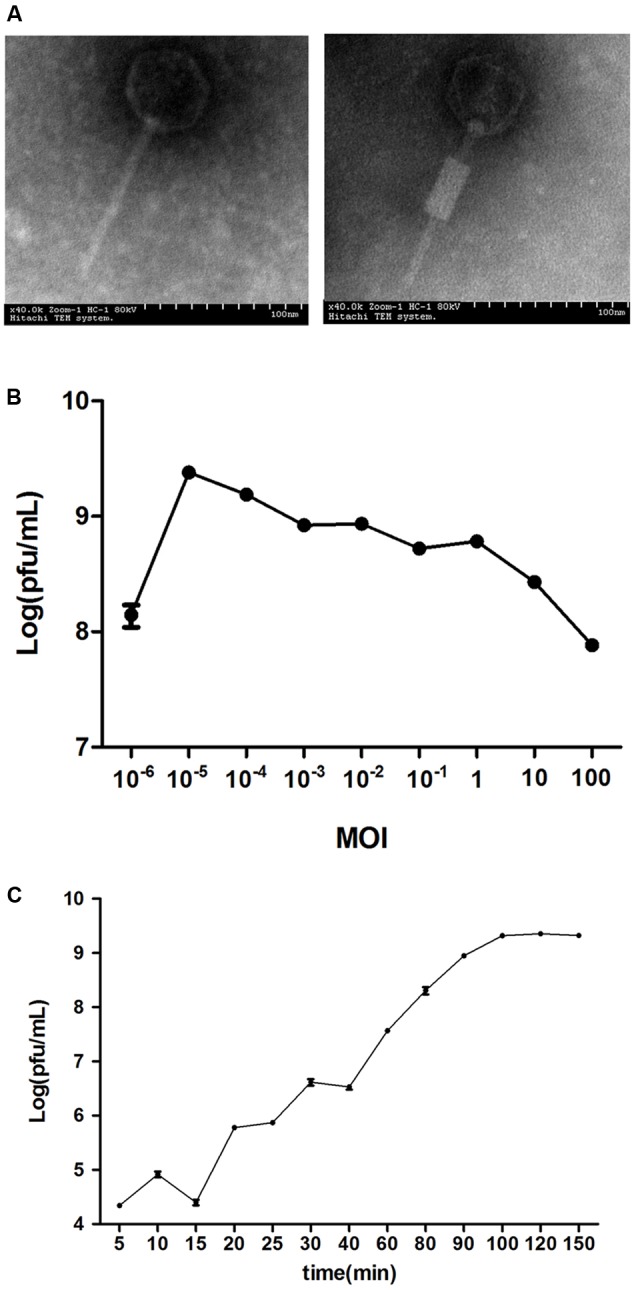
The general characteristics of vB_EcoM_ECOO78. **(A)** The morphology of phage vB_EcoM_ECOO78, as revealed by transmission electron microscope (scale bar: 100 nm). **(B)** Determination of the multiplicity of infection for this bacteriophage. The *x*-axis indicates different MOIs, and the *y*-axis indicates phage titre. Each dot on the graph represents a mean titre. **(C)** One-step growth curve for vB_EcoM_ECOO78. vB_EcoM_ECOO78 was added at an MOI of 10^-5^, and culture samples were harvested at regular intervals. The *x*-axis indicates the time post-infection, and the *y*-axis indicates phage titres. Each dot on the graph represents a mean titre.

The clinical isolates were resistant to a variety of antibiotics, such as quinolones (67.6%), β-lactam antibiotics (76.5%) and aminoglycosides (58.8%) (Supplementary Table S1). The lytic phage vB_EcoM_ECOO78 was isolated successfully from sewage samples by using *E. coli* O78-3 as the host bacterium. vB_EcoM_ECOO78 lysed five out of the 34 tested clinical isolates of pathogenic *E. coli*, including *E. coli* O78-3 (the host strain) (**Table 1**). After prolonged incubation, the areas surrounding the phage plaques developed haloes that increased in size over the course of 96 h (**Figure 1**). The phage had an isometrically hexagonal head of 47 ± 2 nm in diameter. The head was separated from the tail sheath by a collar. The tail was contractile and approximately 149 ± 3 nm long. Based on these morphological characteristics, this phage is a member of the family *Myoviridae* (International Committee on Taxonomy of Viruses) (**Figure 2A**). The highest phage titre was observed at an MOI of 10^-5^ (**Figure 2B**). In the one-step growth curve analysis for the assessment of the infection process, vB_EcoM_ECOO78 had a latent period of approximately 60 min and a burst size of approximately 74 plaque forming units (PFU)/infection (**Figure 2C**).

**Table 1:** The lytic spectrum of vB_EcoM_ECOO78 and the antibiofilm activity of Dpo42.

Bacterial strains	vB_EcoM_ ECOO78^3^	Dpo42^3^	Bacterial strains	vB_EcoM_ ECOO78^3^	Dpo42^3^
*E. coli* HXM^1^	N	Y	*E. coli* O78^1^	Y	Y
*E. coli* ZLH^1^	N	N	*E. coli* 11^1^	Y	Y
*E. coli* LGL^1^	N	N	*E. coli* 6E^1^	Y	Y
*E. coli* ZQB^1^	N	N	*E. coli* 75^1^	Y	Y
*E. coli* DDF^1^	N	N	*E. coli* 45^1^	Y	Y
*E. coli* LSZ^1^	N	Y	*E. coli* 48^1^	N	Y
*E. coli* CZ^1^	N	N	*E. coli* 95^1^	N	Y
*E. coli* ZLH^1^	N	N	*E. coli* 46^1^	N	Y
*E. coli* WZ^1^	N	N	*E. coli* 84^1^	N	Y
*E. coli* YFX^1^	N	N	*E. coli* 9^1^	N	Y
*E. coli* GYP^1^	N	N	*E. coli* 51^1^	N	Y
*E. coli* TSQ^1^	N	N	*E. coli* ATCC 25922^2^	N	N
*E. coli* HSH^1^	N	Y	*E. coli* SDY^1^	N	N
*E. coli* YGZ^1^	N	N	*E. coli* WXR^1^	N	N
*E. coli* WYM^1^	N	N	*E. coli* WQH^1^	N	Y
*E. coli* ZDY^1^	N	N	*E. coli* SGF^1^	N	N
*E. coli* ZYX^1^	N	N	*E. coli* LSR^1^	N	N

*^1^Isolated from patients at the First Hospital of Jilin University (Changchun, Jilin province, China); ^2^Purchased from the ATCC; ^3^Y, yes; N, no.*

### General Features of the vB_EcoM_ECOO78 Genome

**FIGURE 3:**
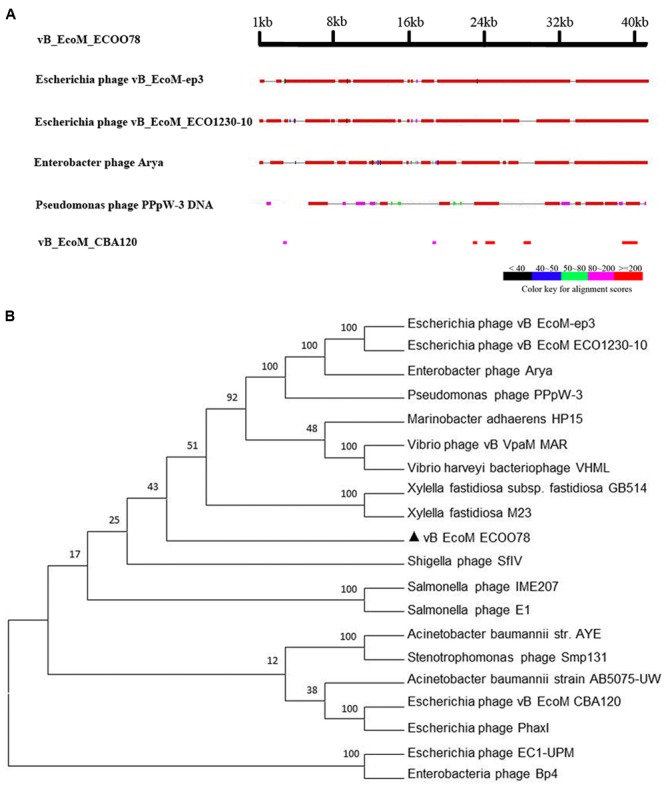
**(A)** A general comparison of the genome of vB_EcoM_ECOO78 with other phages. **(B)** Phylogenetic tree analysis of vB_EcoM_ECOO78 based on terminase large subunit sequences. The numbers next to the branches are bootstrap value and represent confidence (%).

The genome sequence of vB_EcoM_ECOO78 was deposited in GenBank under accession number KY705409. The complete genome of the bacteriophage vB_EcoM_ECOO78 was 41,289 bp with the following nucleotide composition: A (21.57%), T (25.35%), G (25.74%) and C (27.33%). The GC content was 53.07%, and the genome contained both structural and non-structural genes. Based on a comparative analysis of the complete genome, five phages showed similarity to vB_EcoM_ECOO78, specifically vB_EcoM-ep3 (Lv et al., 2015), vB_EcoM_ECO1230-10 (Santos and Bicalho, 2011), *Enterobacter* phage Arya (unpublished), *Pseudomonas* phage PPpW-3 (Kawato et al., 2015) and vB_EcoM_CBA120 (unpublished) (**Figure 3A** and **Table 2**). vB_EcoM_ECOO78 contained 56 predicted ORFs, 51 (91.07%) of which were assumed to be functional. The coordinates and best matches for the sequences are shown in Supplementary Table S2. The remaining five ORFs showed no sequence identity to any of the sequences in the database. As seen from the phylogenetic trees reconstructed based on the terminase large subunit (**Figure 3B**), vB_EcoM_ECOO78 has higher homology with vB_EcoMep3, vB_EcoM_ECO1230-10, *Pseudomonas* phage PPpW-3 and *Enterobacter* phage Arya at the same evolutionary level. *Shigella* phage SfIV, *Salmonella* phage E1, *Salmonella* phage IME207, *Stenotrophomonas* phage Smp131, *Enterobacteria* phage Bp4, *Escherichia* phage EC1-UPM, *Escherichia* phage PhaxI, *Escherichia* phage vB EcoM CBA120, *Vibrio* phage vB VpaM MAR and *Vibrio harveyi* bacteriophage VHML are more distantly related.

**Table 2:** Global phage genome comparison.

	vB_EcoM_ ECOO78	*Escherichia* phage vB_EcoM-ep3	*Escherichia* phage vB_EcoM_ ECO1230-10	*Enterobacter* phage Arya	*Pseudomonas* phage PPpW-3	vB_EcoM_ CBA120
Host strain type	*Escherichia coli*	*Escherichia coli*	*Escherichia coli*	*Enterobacter* sp. *CT7*	*Pseudomonas plecoglossicida*	*Escherichia coli*
GenBank number	KY705409	KM360178.1	GU903191.1	KX231828.1	AB775548.1	JN593240.1
GC content (%)	53.07	53.35	53.37	54.11	61.1	44.5
Genome size (bp)	41,289	42,351	41,666	41,918	43,564	157,304
Identity (BLASTN/ emboss stretcher)	100%/100%	98%/47.8%	94%/48.3%	80%/47.9%	71%/47.8%	67%/21.0%
Query coverage	100%	89%	81%	76%	43%	2%

**FIGURE 4:**
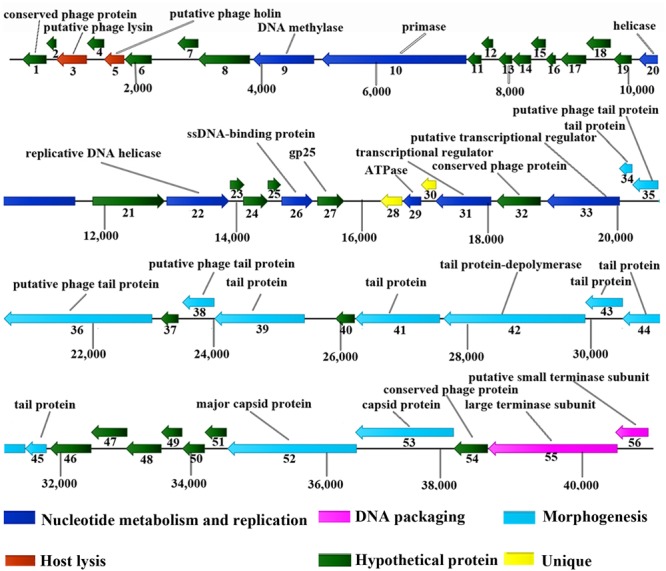
Genomic map of vB_EcoM_ECOO78. Fifty-six open ORFs are presented as arrows; the direction of each arrow represents the direction of transcription. Proposed modules are based on hypothetical functions predicted from bioinformatic analysis.

Approximately 90.2% of ORFs (46 ORFs) started with an ATG codon, 7.8% (4 ORFs) used GTG and only one ORF started with TTG. TGA and TAA were stop codons for 27 (52.9%) and 22 (43.1%) ORFs, respectively. Only ORF48 and ORF53 had TAG as a stop codon. The vB_EcoM_ECOO78 genome is modular (**Figure 4**). Based on bioinformatics predictions, vB_EcoM_ECOO78 encoded a cluster of proteins involved in DNA replication and phage metabolism, including ORF09 (DNA methylase), ORF10 (primase), ORF22 (replicative DNA helicase), ORF26 (single-stranded DNA-binding protein), ORF29 (ATPase), ORF31 (transcriptional regulator) and ORF33 (putative transcriptional regulator). The lysin (ORF03) and holin (ORF05) sequences are important for phage lysis and had ORF sizes of 489 and 324 bp, respectively. The DNA packaging proteins included ORF55 (large terminase subunit) and ORF56 (putative small terminase subunit). The vB_EcoM_ECOO78 genome also encoded proteins associated with tail-baseplate-head morphogenesis, such as ORF34 (tail protein), ORF35 (putative phage tail protein), ORF36 (putative phage tail protein), ORF38 (putative phage tail protein), ORF39 (tail protein), ORF41 (tail protein), ORF43 (tail protein), ORF44 (tail protein), ORF45 (tail protein), ORF52 (major capsid protein) and ORF53 (capsid protein).

### Analysis of Predicted Genes Encoding an EPS Depolymerase

**FIGURE 5:**
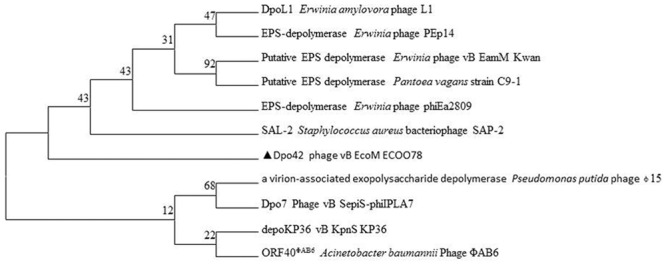
Phylogenetic relationships based on the protein sequences for Dpo42 and 10 other phage depolymerases.

PSI-BLAST indicated that ORF42 demonstrated homology to certain depolymerases, implying that ORF42 might have a function related to EPS synthesis. However, this ORF showed low identity (≤31.0%) to other phage depolymerases (Supplementary Table S3). Dpo42 contained 747 amino acids with a molecular weight of 78.58 KDa and a theoretical pI of 4.77. The secondary structure of Dpo42 was predicted using Phyre2 (Supplementary Figure S1) and included nine α-helices, 55 β-strands and disordered regions. Unfortunately, although the model confidence is 98.0%, due to the low coverage (9%) and identity (45%) with other homologous proteins (including C4ojpC), the modeling failed. As shown in the protein-level phylogenetic tree (**Figure 5**), Dpo42 (black triangle) represents a different evolutionary offshoot branch from the 10 other similar phage depolymerases.

### Dpo42 Antibiofilm Activity

**FIGURE 6:**
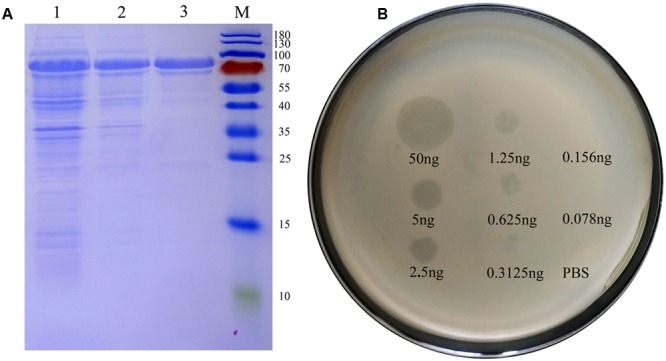
Dpo42 overexpression and activity. **(A)** An SDS-PAGE analysis of purified Dpo42. Lanes: M, protein markers; 1, the induced *E. coli* BL21 cells; 2, supernatant of the induced *E. coli* BL21 cells; 3, purified Dpo42. **(B)** Dpo42 activity against its natural substrate. Serial dilutions of Dpo42 were dropped on the host strain. Elution buffer diluted in PBS was used as a control.

The predicted size of Dpo42 was ∼78.58 KDa, and the protein was expressed and purified (1 μg/μL) from supernatant liquid (**Figure 6A**). Though Dpo42 didn’t show a lytic activity against the *E. coli* (data not shown), Dpo42 prevented the biofilm formation of 15 clinical *E. coli* isolates (**Table 1**). In spot tests, purified Dpo42 (0.1 μg) prevented the biofilm formation of *E. coli* O78-3, and progressive semi-clear spot formation was observed on the plate. Spot size constantly increased after prolonged incubation until 96 h (Supplementary Figure S2). We subsequently dropped serial dilutions of Dpo42 onto the host strain (**Figure 6B**). As the concentration of Dpo42 decreased, the sizes of the semi-clear circles on the plate also decreased. When the concentration of Dpo42 decreased to 0.156 ng, the size of the semi-clear spot resembled that of the negative control.

**FIGURE 7:**
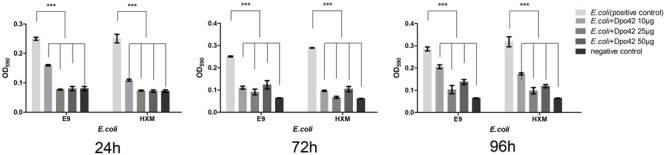
The preventative effect of Dpo42 on biofilm formation. Different doses of Dpo42 or elution buffer diluted in PBS were added to the wells of a 96-well micro-titre plate that contained *E. coli* E9 or HXM strains. The biofilm formation of different groups was detected at 24, 72, and 96 h. ^∗∗∗^*P* < 0.001. Error bars = ± SD; *n* = 3.

**FIGURE 8:**
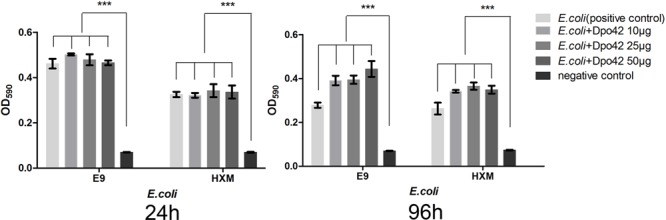
The biofilm removing activity of Dpo42. The E9 and HXM strains formed strong biofilms at 24 or 96 h. The biofilms were treated with different doses of Dpo42. ^∗∗∗^*P* < 0.001. Error bars = ± SD; *n* = 3.

The enzymatic activity of Dpo42 was further tested against clinical *E. coli* strains using 96-well micro-titre plates. Compared with the negative control, the E9 and HXM strains formed strong biofilms (4 × ODc ≤ OD) at 24, 72, and 96 h. By contrast, three groups containing Dpo42 only formed weak biofilms (ODc < OD ≤ 2 × ODc). The OD_590_ values of three groups containing Dpo42 were much lower than that of the positive control (*P* < 0.001; **Figure 7**). Moreover, the OD_590_ of the 50 μg of Dpo42 group was lower than that of the 10 μg of Dpo42 group. It indicated that Dpo42 can prevent the formation of the biofilm and exhibited dose-dependent activity during 96 h. Additionally, 24 and 96 h old biofilms were treated with different concentrations of Dpo42 (10 μg/well, 25 μg/well, 50 μg/well). The strong biofilms (4 × ODc ≤ OD) formed at 24 and 96 h by E9 and HXM strains couldn’t be destroyed by Dpo42. The OD_590_ values of three groups treated with Dpo42 were much higher than that of the negative control (*P* < 0.001; **Figure 8**). Thus, Dpo42 can only prevent the formation of biofilm, but can not remove it.

**FIGURE 9:**
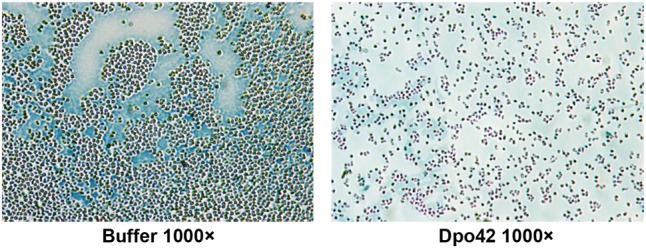
Capsule staining of *E. coli* E9 in the absence and presence of Dpo42. Capsules were negatively stained with Maneval’s solution and appeared white underneath the microscope.

Staining assays indicated that *E. coli* adhered to each other and were encircled by a white capsule. By contrast, cells were separated and almost lost the white capsule after treatment with Dpo42 (**Figure 9**). Thus, the target of Dpo42 belongs to the capsular polysaccharides.

## Discussion

In this study, vB_EcoM_ECOO78 was isolated and observed to induce the formation of increasing halos surrounding the lysis plaques. This phenomenon was presumably caused by a phage-related depolymerase (Harper et al., 2014; Pires et al., 2016). Depolymerases have multiple applications for the prevention or eradication of biofilms (Hughes et al., 1998b; Verma et al., 2010) and may be useful as therapeutic agents against bacterial pathogens (Mushtaq et al., 2004; Scorpio et al., 2008). To identify the depolymerase of vB_EcoM_ECOO78, the complete genome of this phage was sequenced and analyzed. The vB_EcoM_ECOO78 genome was modular, which is common among phages (Veesler and Cambillau, 2011). A comparative analysis indicated that five phages, specifically vB_EcoM-ep3 (Lv et al., 2015), vB_EcoM_ECO1230-10 (Santos and Bicalho, 2011), *Enterobacter* phage Arya (unpublished), *Pseudomonas* phage PPpW-3 (Kawato et al., 2015) and vB_EcoM_CBA120 (unpublished), showed similarity to vB_EcoM_ECOO78. However, there were certain genes in vB_EcoM_ECOO78 that shared no identity with these similar phages. Furthermore, three ORFs, specifically ORF03 (lysin), ORF39 (tail protein) and ORF53 (capsid protein), were related to *E. coli* APEC O2, *Haematospirillum jordaniae* and *Xylella fastidiosa* MUL0034, respectively. Genetic drift and recombination between different phages, as well as between vB_EcoM_ECOO78 and bacteria, were evident for certain ORFs.

Based on our bioinformatics predictions, the ORF42 of vB_EcoM_ECOO78 (Dpo42) most likely encodes a depolymerase. Interestingly, Dpo42 showed low identity (≤31.0%) to other phage depolymerases. Thus, Dpo42 is a novel depolymerase from an *E. coli* phage. The *Klebsiella* phage depolymerase depoKP36 reportedly contains a high β-sheet content. This was similar to Dpo42, which contains 55 β-sheets. Given our observation of the similarity between Dpo42 and the tail proteins of other phages, Dpo42 is likely a tail-related depolymerase.

To further characterise the activity of Dpo42, this depolymerase was expressed in *E. coli*. Dpo42 expression did not affect *E. coli* BL21. However, Dpo42 did demonstrate biofilm-formation prevention activity against several *E. coli* isolates *in vitro*. In addition, dose-dependent activity was observed when *E. coli* biofilms were treated with Dpo42 during 96 h. The bacterial EPS-degrading abilities of phage depolymerases have been reported for more than 70 years (Zobell, 1943; Bartell et al., 1966). To the best of our knowledge, there have only been three *E. coli* phage-derived depolymerases purified from *E. coli* phage lysates, and all were able to degrade the capsular layers on host cells (Tomlinson and Taylor, 1985; McCallum et al., 1989; Petter and Vimr, 1993). In addition, to the best of our knowledge, Dpo42 is the first genetically engineered phage-derived depolymerase with the ability to prevent *E. coli* biofilm formation. Notably, Dpo42 shows no similarity to the three previously reported *E. coli* phage-derived depolymerases. Among previously described depolymerases, only DepoKP36 has demonstrated a broad depolymerization range, hydrolysing the EPS formed Sby 8/11 *Klebsiella* isolates (Majkowska-Skrobek et al., 2016). In this study, Dpo42 prevented biofilm formation of 15/34 *E. coli* isolates and the target of Dpo42 was capsular polysaccharide.

Although most bacterial biofilms are composed of EPS, they also contain water, proteins, lipids, nucleic acids, and mineral ions (Coenye and Nelis, 2010; Flemming and Wingender, 2010). The residual proteins and nucleic acids may block depolymerases from eradicating the biofilms completely. To our knowledge, only phage-derived depolymerase Dpo7 has been proven to be effective against polysaccharidic matrixes of biofilms formed by *Staphylococcus epidermidis* and *Staphylococcus aureus* (Gutierrez et al., 2015). Dpo42 was unable to remove young or mature biofilms. We conclude that Dpo42 may have its effect on PGA, in consideration that PGA serves in the initial adhesion, which is the first step involved in biofilm formation (Sharma et al., 2016).

It may be possible to genetically modify depolymerases to amplify enzyme activity or mix different depolymerases to create a therapeutic cocktail (Yen et al., 2017). Bansal et al. (2014) evaluated the activity of depoKP36 in combination with four classes of antibiotics, specifically ciprofloxacin (a fluoroquinolone), oxytetracycline (a tetracycline), gentamicin and chloramphenicol, and observed synergistic effects for the combination of depoKP36 and gentamicin, as well as improved gentamicin activity. Thus, combination therapy employing antibiotics and a depolymerase may represent a more efficient treatment strategy for bacterial infections.

## Author Contributions

ZG, JG, and JY drafted the main manuscript and performed the data analysis; ZG, JH, GY, LL, SW, LY, LZ, AG, XF, WH, JG, and JY planned and performed experiments; ZG, JG, LL, and JY were responsible for experimental design; and ZG, JY, and JG were responsible for guiding and supporting the experiments and manuscript revisions.

## Conflict of Interest Statement

The authors declare that the research was conducted in the absence of any commercial or financial relationships that could be construed as a potential conflict of interest.

## Footnotes

1 http://bioinf.ibun.unal.edu.co/servicios/sms/orf_find.html

2 http://ccb.jhu.edu/software/glimmer/index.shtml

3 https://blast.ncbi.nlm.nih.gov/

4 http://pfam.xfam.org/family

5 http://www.ebi.ac.uk/Tools/sss/psiblast

6 http://www.sbg.bio.ic.ac.uk/phyre2
