# Corrigendum: Identification and Characterization of Dpo42, a Novel Depolymerase Derived from the *Escherichia coli* Phage vB_EcoM_ECOO78

**DOI:** 10.3389/fmicb.2017.02103

**Published:** 2017-10-25

**Authors:** Zhimin Guo, Jing Huang, Guangmou Yan, Liancheng Lei, Shuang Wang, Ling Yu, Liang Zhou, Anchong Gao, Xin Feng, Wenyu Han, Jingmin Gu, Junling Yang

**Affiliations:** ^1^Department of Respiratory Medicine, The Second Hospital of Jilin University, Changchun, China; ^2^Department of Clinical Laboratory, The First Hospital of Jilin University, Changchun, China; ^3^Key Laboratory of Zoonosis Research, Ministry of Education, College of Veterinary Medicine, Jilin University, Changchun, China; ^4^Agricultural Experiment Base, Jilin University, Changchun, China

**Keywords:** bacteriophage, depolymerase, *Escherichia coli*, biofilm, beta-lactamase

In the original article, there was a mistake in the FUNDING section as published. The National Key Basic Research Program of China (No. 2013CB127205) was the primary funding and should be listed as the first one.

The corrected FUNDING appears below:

This work was supported by grants from the National Key Basic Research Program of China (No. 2013CB127205), National Natural Science Foundation of China (No. 31572553 and 31502103), the National Key Research and Development Program of China (No. 2017YFD0501000), and the Development Program of the First Hospital of Jilin University (No. JDYY82017022).

The authors apologize for this error and state that this does not change the scientific conclusions of the article in any way.

## Conflict of interest statement

The authors declare that the research was conducted in the absence of any commercial or financial relationships that could be construed as a potential conflict of interest.

