# Supplementary material for: Identification and Characterization of Dpo42, a Novel Depolymerase Derived from the Escherichia coli Phage vB_EcoM_ECOO78
Source: Front Microbiol. 2017 Aug 2;8:1460. doi: 10.3389/fmicb.2017.01460 (PMC5539073; doi:10.3389/fmicb.2017.01460)
Supplement: Supplementary file 1 [file Table_1.DOC]

**Table S1. The antibiotic resistance of *E. coli* isolates.**

| *E. coli* | Cefuroxime | Piperacillin | Ceftriaxone | Ceftazidime | Cefepime | Piperacillin/Tazobactam | Ampicillin/sulbactam | Levofloxacin | Ciprofloxacin | Gentamicin | Amikacin | Sulfamethoxazole trimethoprim | Meropenem |
| --- | --- | --- | --- | --- | --- | --- | --- | --- | --- | --- | --- | --- | --- |
| ATCC25922^2^ | S | S | S | S | S | S | S | S | S | S | S | S | S |
| O78^1^ | S | S | S | S | S | S | S | S | S | R | S | S | S |
| 11^1^ | S | S | S | S | S | S | S | R | R | R | S | S | S |
| 6E1 | R | S | S | S | S | S | S | S | S | R | R | S | S |
| 75^1^ | R | R | S | S | S | S | R | S | S | S | S | S | S |
| 45^1^ | R | R | R | R | S | S | R | R | R | R | S | R | S |
| 48^1^ | R | R | S | S | S | S | R | S | S | S | S | S | S |
| 95^1^ | R | R | R | R | S | S | R | R | R | S | S | R | S |
| 46^1^ | R | R | R | R | S | S | R | R | R | R | S | R | S |
| 84^1^ | R | S | S | S | S | S | S | S | S | R | R | S | S |
| 9^1^ | R | R | R | R | S | S | R | R | R | S | S | R | S |
| 52^1^ | R | I | S | S | S | S | R | R | R | R | S | R | S |
| HXM^1^ | R | R | R | R | I | S | R | R | R | R | S | R | S |
| WYM^1^ | I | I | S | S | S | S | I | S | I | S | S | R | S |
| ZLH^1^ | R | I | S | S | S | S | R | R | R | R | S | R | S |
| LGL^1^ | R | R | S | S | S | S | R | S | I | S | S | S | S |
| ZQB^1^ | R | R | S | S | S | S | R | S | S | S | S | S | S |
| DDF^1^ | R | R | R | R | S | S | R | R | R | S | S | R | S |
| LSZ^1^ | R | R | R | R | S | S | R | R | R | R | S | R | S |
| CZ^1^ | S | R | S | S | S | S | R | I | R | R | S | R | S |
| ZLH^1^ | R | I | S | S | S | S | R | R | R | R | S | R | S |
| WZ^1^ | R | R | R | S | S | S | R | R | R | R | S | S | S |
| YFX^1^ | R | I | R | S | S | S | S | R | R | R | S | R | S |
| GYP^1^ | S | S | S | S | S | S | R | R | R | S | S | R | S |
| TSQ^1^ | R | R | R | R | S | S | R | R | R | R | S | R | S |
| HSH^1^ | R | R | R | S | S | S | R | S | S | S | S | R | S |
| YGZ^1^ | R | R | R | S | S | S | R | R | R | R | S | R | S |
| ZDY^1^ | R | R | R | S | S | S | R | R | R | R | S | R | S |
| ZYX^1^ | R | R | R | R | I | S | R | R | R | S | S | R | S |
| SGF^1^ | I | I | S | S | S | S | R | S | S | S | S | S | S |
| LSR^1^ | R | R | R | R | R | I | R | R | R | R | I | R | S |
| SDY^1^ | R | R | R | R | R | S | R | R | R | R | R | R | S |
| WXR^1^ | R | R | R | R | R | S | R | R | R | R | R | R | S |
| WQH^1^ | R | R | R | R | R | S | R | R | R | S | S | R | S |

S, Susceptible; I, Intermediate; R, Resistance. ^1^ Isolated from patients at the First Hospital of Jilin University (Changchun, Jilin province, China); ^2^ purchased from the American Type Culture Collection (ATCC).
