# Supplementary material for: Identification and Characterization of Dpo42, a Novel Depolymerase Derived from the Escherichia coli Phage vB_EcoM_ECOO78
Source: Front Microbiol. 2017 Aug 2;8:1460. doi: 10.3389/fmicb.2017.01460 (PMC5539073; doi:10.3389/fmicb.2017.01460)
Supplement: Supplementary file 2 [file Table_2.DOC]

**Table S2.** Descriptions of the predicted ORFs and functions of vB_EcoM_ECOO78.

| ORFs^a^ | Start | End | Length(aa) | Molecular mass(KD) | Predicted function | Best homolog | Query cover | E  value | Identity  (positives) | Accession no. |
| --- | --- | --- | --- | --- | --- | --- | --- | --- | --- | --- |
| ORF01- | 205 | 591 | 128 | 14.33 | conserved phage protein | [[](https://blast.ncbi.nlm.nih.gov/Blast.cgi#alnHdr_937533311)*Escherichia* phage vB_EcoM_ECO1230-10] | 83% | 3e-27 | 66% | [YP_009168914.1](https://www.ncbi.nlm.nih.gov/protein/937533311?report=genbank&log$=prottop&blast_rank=1&RID=BPHTAZ3D013) |
| ORF02- | 588 | 749 | 53 | 6.38 | hypothetical protein SEEM030_05686 | [[](https://blast.ncbi.nlm.nih.gov/Blast.cgi#alnHdr_363569009)*Salmonella enterica* subsp. enterica serovar *Montevideo* str. SARB30] | 94% | 4e-11 | 60% | [EHL52975.1](https://www.ncbi.nlm.nih.gov/protein/363569009?report=genbank&log$=prottop&blast_rank=1&RID=BPJ53RY4013) |
| ORF03- | 746 | 1234 | 162 | 17.61 | putative phage lysin | [[](https://blast.ncbi.nlm.nih.gov/Blast.cgi#alnHdr_937533312)*Escherichia* phage vB_EcoM_ECO1230-10] | 100% | 1e-111 | 98% | [YP_009168915.1](https://www.ncbi.nlm.nih.gov/protein/937533312?report=genbank&log$=prottop&blast_rank=1&RID=BPJ5GC8A013) |
| ORF04- | 1237 | 1512 | 91 | 10.21 | hypothetical protein | [[](https://blast.ncbi.nlm.nih.gov/Blast.cgi#alnHdr_937533311)*Escherichia* phage vB_EcoM_ECO1230-10] | 100% | 5e-57 | 97% | [YP_009168916.1](https://www.ncbi.nlm.nih.gov/protein/937533313?report=genbank&log$=prottop&blast_rank=1&RID=BPJAMEH2013) |
| ORF05- | 1509 | 1832 | 107 | 11.64 | putative phage holin | [[](https://blast.ncbi.nlm.nih.gov/Blast.cgi#alnHdr_937533311)*Escherichia* phage vB_EcoM_ECO1230-10] | 100% | 7e-72 | 100% | [YP_009168917.1](https://www.ncbi.nlm.nih.gov/protein/937533314?report=genbank&log$=prottop&blast_rank=1&RID=BPK2PWTW016) |
| ORF06- | 1838 | 2272 | 144 | 16.71 | hypothetical protein | [[](https://blast.ncbi.nlm.nih.gov/Blast.cgi#alnHdr_937533311)*Escherichia* phage vB_EcoM_ECO1230-10] | 100% | 8e-99 | 95% | [YP_009168918.1](https://www.ncbi.nlm.nih.gov/protein/937533315?report=genbank&log$=prottop&blast_rank=1&RID=BPK34H6N013) |
| ORF07- | 2685 | 3017 | 110 | 12.60 | hypothetical protein | [[](https://blast.ncbi.nlm.nih.gov/Blast.cgi#alnHdr_712913194)*Escherichia* phage vB_EcoM-ep3] | 95% | 3e-65 | 93% | [YP_009100014.1](https://www.ncbi.nlm.nih.gov/protein/712913192?report=genbank&log$=prottop&blast_rank=1&RID=BPKG9XN1013) |
| ORF08- | 3014 | 3841 | 275 | 31.02 | hypothetical protein | [[](https://blast.ncbi.nlm.nih.gov/Blast.cgi#alnHdr_712913194)*Escherichia* phage vB_EcoM-ep3] | 100% | 0.0 | 99% | [YP_009100013.1](https://www.ncbi.nlm.nih.gov/protein/712913191?report=genbank&log$=prottop&blast_rank=1&RID=BPKGPSB4013) |
| ORF09- | 3889 | 4866 | 325 | 36.55 | DNA methylase | [[](https://blast.ncbi.nlm.nih.gov/Blast.cgi#alnHdr_712913194)*Escherichia* phage vB_EcoM-ep3] | 100% | 0.0 | 99% | [YP_009100012.1](https://www.ncbi.nlm.nih.gov/protein/712913190?report=genbank&log$=prottop&blast_rank=1&RID=BPM070U2016) |
| ORF10- | 4990 | 7299 | 769 | 86.39 | primase | [[](https://blast.ncbi.nlm.nih.gov/Blast.cgi#alnHdr_712913194)*Escherichia* phage vB_EcoM-ep3] | 100% | 0.0 | 98% | [YP_009100011.1](https://www.ncbi.nlm.nih.gov/protein/712913189?report=genbank&log$=prottop&blast_rank=1&RID=BPM0MFSP016) |
| ORF11- | 7312 | 7542 | 76 | 8.78 | hypothetical protein | [[](https://blast.ncbi.nlm.nih.gov/Blast.cgi#alnHdr_937533311)*Escherichia* phage vB_EcoM_ECO1230-10] | 100% | 3e-46 | 97% | [YP_009168924.1](https://www.ncbi.nlm.nih.gov/protein/937533321?report=genbank&log$=prottop&blast_rank=1&RID=BPMFN794013) |
| ORF12- | 7539 | 7727 | 62 | 7.07 | hypothetical protein | [[](https://blast.ncbi.nlm.nih.gov/Blast.cgi#alnHdr_712913194)*Escherichia* phage vB_EcoM-ep3] | 100% | 3e-36 | 98% | [YP_009100009.1](https://www.ncbi.nlm.nih.gov/protein/712913187?report=genbank&log$=prottop&blast_rank=1&RID=BPMG1V04013) |
| ORF13- | 7808 | 8032 | 74 | 8.05 | hypothetical protein | [[](https://blast.ncbi.nlm.nih.gov/Blast.cgi#alnHdr_712913194)*Escherichia* phage vB_EcoM-ep3] | 100% | 1e-46 | 99% | [YP_009100008.1](https://www.ncbi.nlm.nih.gov/protein/712913186?report=genbank&log$=prottop&blast_rank=1&RID=BPMVHCXG013) |
| ORF15- | 8332 | 8568 | 78 | 9.03 | hypothetical protein | [[](https://blast.ncbi.nlm.nih.gov/Blast.cgi#alnHdr_937533311)*Escherichia* phage vB_EcoM_ECO1230-10] | 98% | 1e-44 | 94% | [YP_009168928.1](https://www.ncbi.nlm.nih.gov/protein/937533325?report=genbank&log$=prottop&blast_rank=1&RID=BPN29RCM01N) |
| ORF16- | 8565 | 8729 | 54 | 6.28 | hypothetical protein | [[](https://blast.ncbi.nlm.nih.gov/Blast.cgi#alnHdr_937533311)*Escherichia* phage vB_EcoM_ECO1230-10] | 100% | 6e-29 | 94% | [YP_009168929.1](https://www.ncbi.nlm.nih.gov/protein/937533326?report=genbank&log$=prottop&blast_rank=1&RID=BPN2NJTP01N) |
| ORF17- | 8805 | 9215 | 136 | 14.63 | hypothetical protein | [[](https://blast.ncbi.nlm.nih.gov/Blast.cgi#alnHdr_712913194)*Escherichia* phage vB_EcoM-ep3] | 100% | 5e-93 | 99% | [YP_009100005.1](https://www.ncbi.nlm.nih.gov/protein/712913183?report=genbank&log$=prottop&blast_rank=1&RID=BPN31SPX01N) |
| ORF18- | 9215 | 9607 | 130 | 14.50 | hypothetical protein | [[](https://blast.ncbi.nlm.nih.gov/Blast.cgi#alnHdr_712913194)*Escherichia* phage vB_EcoM-ep3] | 98% | 1e-88 | 98% | [YP_009100004.1](https://www.ncbi.nlm.nih.gov/protein/712913182?report=genbank&log$=prottop&blast_rank=1&RID=BPPFR71J01N) |
| ORF20- | 10052 | 11467 | 471 | 51.71 | helicase | [[](https://blast.ncbi.nlm.nih.gov/Blast.cgi#alnHdr_712913194)*Escherichia* phage vB_EcoM-ep3] | 100% | 0.0 | 99% | [YP_009100002.1](https://www.ncbi.nlm.nih.gov/protein/712913180?report=genbank&log$=prottop&blast_rank=1&RID=BPNUE0W101N) |
| ORF21+ | 11741 | 12883 | 380 | 42.41 | PDDEXK-like domain of unknown function | [[](https://blast.ncbi.nlm.nih.gov/Blast.cgi#alnHdr_712913194)*Escherichia* phage vB_EcoM-ep3] | 100% | 0.0 | 99% | [YP_009100001.1](https://www.ncbi.nlm.nih.gov/protein/712913179?report=genbank&log$=prottop&blast_rank=1&RID=BPR3TH9V01N) |
| ORF22+ | 12911 | 13900 | 329 | 36.54 | replicative DNA helicase | [[](https://blast.ncbi.nlm.nih.gov/Blast.cgi#alnHdr_712913194)*Escherichia* phage vB_EcoM-ep3] | 100% | 0.0 | 99% | [YP_009100000.1](https://www.ncbi.nlm.nih.gov/protein/712913181?report=genbank&log$=prottop&blast_rank=1&RID=BPR3DR6501N) |
| ORF23+ | 13914 | 14141 | 75 | 8.22 | hypothetical protein | [[](https://blast.ncbi.nlm.nih.gov/Blast.cgi#alnHdr_712913194)*Escherichia* phage vB_EcoM-ep3] | 98% | 4e-48 | 100% | [YP_009099999.1](https://www.ncbi.nlm.nih.gov/protein/712913178?report=genbank&log$=prottop&blast_rank=1&RID=BPPTGBUN01N) |
| ORF24+ | 14122 | 14517 | 131 | 14.16 | hypothetical protein | [[](https://blast.ncbi.nlm.nih.gov/Blast.cgi#alnHdr_712913194)*Escherichia* phage vB_EcoM-ep3] | 100% | 4e-91 | 99% | [YP_009099998.1](https://www.ncbi.nlm.nih.gov/protein/712913177?report=genbank&log$=prottop&blast_rank=1&RID=BPRMX1AX013) |
| ORF26+ | 14728 | 15225 | 165 | 18.57 | ssDNA-binding protein | [[](https://blast.ncbi.nlm.nih.gov/Blast.cgi#alnHdr_727192951)*Serratia*] | 99% | 2e-53 | 57% | [WP_033654707.1](https://www.ncbi.nlm.nih.gov/protein/727192951?report=genbank&log$=prottop&blast_rank=1&RID=BPRYEJS1013) |
| ORF27+ | 15294 | 15719 | 141 | 16.47 | gp25 | [[](https://blast.ncbi.nlm.nih.gov/Blast.cgi#alnHdr_593780619)*Escherichia* phage EB49] | 82% | 5e-48 | 62% | [YP_009018639.1](https://www.ncbi.nlm.nih.gov/protein/593780619?report=genbank&log$=prottop&blast_rank=1&RID=BPRYVNJ1013) |
| ORF29- | 16646 | 16936 | 96 | 10.51 | ATPase | [[](https://blast.ncbi.nlm.nih.gov/Blast.cgi#alnHdr_1070100414)*Enterobacter* phage Arya] | 100% | 3e-28 | 59% | [YP_009284294.1](https://www.ncbi.nlm.nih.gov/protein/1070100392?report=genbank&log$=prottop&blast_rank=1&RID=BPS00PM4013) |
| ORF31- | 17162 | 18043 | 293 | 31.98 | transcriptional regulator | [[](https://blast.ncbi.nlm.nih.gov/Blast.cgi#alnHdr_712913194)*Escherichia* phage vB_EcoM-ep3] | 98% | 0.0 | 99% | [YP_009100045.1](https://www.ncbi.nlm.nih.gov/protein/712913221?report=genbank&log$=prottop&blast_rank=1&RID=BPSJXWXD01N) |
| ORF32- | 18124 | 18825 | 233 | 25.96 | conserved phage protein | [[](https://blast.ncbi.nlm.nih.gov/Blast.cgi#alnHdr_937533327)*Escherichia* phage vB_EcoM_ECO1230-10] | 100% | 2e-76 | 53% | [YP_009168888.1](https://www.ncbi.nlm.nih.gov/protein/937533285?report=genbank&log$=prottop&blast_rank=1&RID=BPSKJKWJ01N) |
| ORF33- | 18920 | 20074 | 384 | 41.77 | Phage late control gene D protein (GPD) | [[](https://blast.ncbi.nlm.nih.gov/Blast.cgi#alnHdr_937533327)*Escherichia* phage vB_EcoM_ECO1230-10] | 100% | 0.0 | 98% | [YP_009168889.1](https://www.ncbi.nlm.nih.gov/protein/937533286?report=genbank&log$=prottop&blast_rank=1&RID=BPSKX74W01N) |
| ORF34- | 20065 | 20274 | 69 | 7.46 | tail protein | [[](https://blast.ncbi.nlm.nih.gov/Blast.cgi#alnHdr_712913194)*Escherichia* phage vB_EcoM-ep3] | 100% | 4e-41 | 97% | [YP_009100042.1](https://www.ncbi.nlm.nih.gov/protein/712913219?report=genbank&log$=prottop&blast_rank=1&RID=BPT5UGAD01N) |
| ORF35- | 20274 | 20684 | 136 | 15.57 | putative phage tail protein | [[](https://blast.ncbi.nlm.nih.gov/Blast.cgi#alnHdr_937533327)*Escherichia* phage vB_EcoM_ECO1230-10] | 100% | 8e-94 | 97% | [YP_009168891.1](https://www.ncbi.nlm.nih.gov/protein/937533288?report=genbank&log$=prottop&blast_rank=1&RID=BPT646AE01N) |
| ORF36- | 20681 | 23026 | 781 | 82.25 | putative phage tail protein | [*Escherichia* phage vB_EcoM_ECO1230-10] | 100% | 0.0 | 96% | [YP_009168892.1](https://www.ncbi.nlm.nih.gov/protein/937533289?report=genbank&log$=prottop&blast_rank=1&RID=BPUUKPYH013) |
| ORF37- | 23158 | 23442 | 94 | 10.55 | Phage tail assembly chaperone proteins | [[](https://blast.ncbi.nlm.nih.gov/Blast.cgi#alnHdr_712913216)*Escherichia* phage vB_EcoM-ep3] | 100% | 4e-62 | 96% | [YP_009100039.1](https://www.ncbi.nlm.nih.gov/protein/712913216?report=genbank&log$=prottop&blast_rank=1&RID=BPTKX8DH01N) |
| ORF38- | 23503 | 24009 | 168 | 18.08 | Phage tail tube protein FII | [[](https://blast.ncbi.nlm.nih.gov/Blast.cgi#alnHdr_937533327)*Escherichia* phage vB_EcoM_ECO1230-10] | 100% | 4e-120 | 100% | [YP_009168894.1](https://www.ncbi.nlm.nih.gov/protein/937533291?report=genbank&log$=prottop&blast_rank=1&RID=BPTTMU5X01N) |
| ORF39- | 24009 | 25436 | 475 | 49.70 | Phage tail sheath protein | [[](https://blast.ncbi.nlm.nih.gov/Blast.cgi#alnHdr_712913194)*Escherichia* phage vB_EcoM-ep3] | 100% | 0.0 | 97% | [YP_009100037.1](https://www.ncbi.nlm.nih.gov/protein/712913214?report=genbank&log$=prottop&blast_rank=1&RID=BPTZRPKR01N) |
| ORF40- | 25923 | 26228 | 101 | 10.92 | hypothetical protein | [[](https://blast.ncbi.nlm.nih.gov/Blast.cgi#alnHdr_712913194)*Escherichia* phage vB_EcoM-ep3] | 100% | 1e-67 | 100% | [YP_009100035.1](https://www.ncbi.nlm.nih.gov/protein/712913212?report=genbank&log$=prottop&blast_rank=1&RID=BPUW40Y9016) |
| ORF41- | 26237 | 27574 | 445 | 47.38 | Phage tail-collar fibre protein | [[](https://blast.ncbi.nlm.nih.gov/Blast.cgi#alnHdr_712913194)*Escherichia* phage vB_EcoM-ep3] | 100% | 0.0 | 99% | [YP_009100034.1](https://www.ncbi.nlm.nih.gov/protein/712913211?report=genbank&log$=prottop&blast_rank=1&RID=BPV60PBA01N) |
| ORF42- | 27628 | 29871 | 747 | 78.58 | exopolysaccharide depolymerase | [[](https://blast.ncbi.nlm.nih.gov/Blast.cgi#alnHdr_712913194)*Escherichia* phage vB_EcoM-ep3] | 91% | 0.0 | 99% | [YP_009100033.1](https://www.ncbi.nlm.nih.gov/protein/712913210?report=genbank&log$=prottop&blast_rank=1&RID=BPV73S2J01N) |
| ORF43- | 29872 | 30465 | 197 | 22.27 | tail protein | [[](https://blast.ncbi.nlm.nih.gov/Blast.cgi#alnHdr_712913194)*Escherichia* phage vB_EcoM-ep3] | 100% | 2e-143 | 100% | [YP_009100032.1](https://www.ncbi.nlm.nih.gov/protein/712913209?report=genbank&log$=prottop&blast_rank=1&RID=BRBKK6C5013) |
| ORF44- | 30458 | 31360 | 300 | 31.55 | Baseplate J-like protein | [[](https://blast.ncbi.nlm.nih.gov/Blast.cgi#alnHdr_712913194)*Escherichia* phage vB_EcoM-ep3] | 100% | 0.0 | 100% | [YP_009100031.1](https://www.ncbi.nlm.nih.gov/protein/712913208?report=genbank&log$=prottop&blast_rank=1&RID=BRBMEDX4013) |
| ORF45- | 31361 | 31699 | 112 | 12.12 | Gene 25-like lysozyme | [[](https://blast.ncbi.nlm.nih.gov/Blast.cgi#alnHdr_712913194)*Escherichia* phage vB_EcoM-ep3] | 100% | 9e-75 | 100% | [YP_009100030.1](https://www.ncbi.nlm.nih.gov/protein/712913207?report=genbank&log$=prottop&blast_rank=1&RID=BRBN2WJ1013) |
| ORF46- | 31753 | 32406 | 217 | 22.25 | Type VI secretion system, phage-baseplate injector | [[](https://blast.ncbi.nlm.nih.gov/Blast.cgi#alnHdr_712913194)*Escherichia* phage vB_EcoM-ep3] | 100% | 9e-156 | 99% | [YP_009100029.1](https://www.ncbi.nlm.nih.gov/protein/712913206?report=genbank&log$=prottop&blast_rank=1&RID=BRC6JPET013) |
| ORF47- | 32403 | 32975 | 190 | 21.25 | hypothetical protein | [[](https://blast.ncbi.nlm.nih.gov/Blast.cgi#alnHdr_712913194)*Escherichia* phage vB_EcoM-ep3] | 98% | 7e-129 | 96% | [YP_009100028.1](https://www.ncbi.nlm.nih.gov/protein/712913205?report=genbank&log$=prottop&blast_rank=1&RID=BRC6W0G7013) |
| ORF48- | 32951 | 33514 | 187 | 21.50 | hypothetical protein | [[](https://blast.ncbi.nlm.nih.gov/Blast.cgi#alnHdr_1057604131)*Acinetobacter celticus*] | 97% | 4e-43 | 45% | [WP_068888210.1](https://www.ncbi.nlm.nih.gov/protein/1057604131?report=genbank&log$=prottop&blast_rank=1&RID=BRC7E3M0013) |
| ORF49- | 33511 | 33843 | 110 | 11.94 | hypothetical protein | [[](https://blast.ncbi.nlm.nih.gov/Blast.cgi#alnHdr_712913194)*Escherichia* phage vB_EcoM-ep3] | 98% | 9e-73 | 99% | [YP_009100026.1](https://www.ncbi.nlm.nih.gov/protein/712913203?report=genbank&log$=prottop&blast_rank=1&RID=BRC7WAD5013) |
| ORF50- | 33843 | 34199 | 118 | 12.11 | hypothetical protein | [[](https://blast.ncbi.nlm.nih.gov/Blast.cgi#alnHdr_712913194)*Escherichia* phage vB_EcoM-ep3] | 100% | 6e-79 | 99% | [YP_009100025.1](https://www.ncbi.nlm.nih.gov/protein/712913202?report=genbank&log$=prottop&blast_rank=1&RID=BRDK0TZ0013) |
| ORF51- | 34199 | 34546 | 115 | 12.42 | Rho termination factor, N-terminal domain | [[](https://blast.ncbi.nlm.nih.gov/Blast.cgi#alnHdr_712913194)*Escherichia* phage vB_EcoM-ep3] | 86% | 2e-62 | 99% | [YP_009100024.1](https://www.ncbi.nlm.nih.gov/protein/712913201?report=genbank&log$=prottop&blast_rank=1&RID=BRDSXXC6013) |
| ORF52- | 34555 | 36600 | 681 | 73.75 | major capsid protein | [[](https://blast.ncbi.nlm.nih.gov/Blast.cgi#alnHdr_712913194)*Escherichia* phage vB_EcoM-ep3] | 100% | 0.0 | 98% | [YP_009100023.1](https://www.ncbi.nlm.nih.gov/protein/712913200?report=genbank&log$=prottop&blast_rank=1&RID=BRE2XCXW01N) |
| ORF53- | 36578 | 38134 | 518 | 58.53 | Phage portal protein, lambda family | [[](https://blast.ncbi.nlm.nih.gov/Blast.cgi#alnHdr_712913194)*Escherichia* phage vB_EcoM-ep3] | 100% | 0.0 | 98% | [YP_009100022.1](https://www.ncbi.nlm.nih.gov/protein/712913199?report=genbank&log$=prottop&blast_rank=1&RID=BRE8WZGM016) |
| ORF54- | 38134 | 38673 | 179 | 19.88 | conserved phage protein | [*Escherichia* phage vB_EcoM_ECO1230-10] | 100% | 6e-129 | 99% | [YP_009168911.1](https://www.ncbi.nlm.nih.gov/protein/937533308?report=genbank&log$=prottop&blast_rank=1&RID=BRE9BCNX016) |
| ORF55- | 38680 | 40722 | 680 | 76.48 | large terminase subunit | [[](https://blast.ncbi.nlm.nih.gov/Blast.cgi#alnHdr_712913194)*Escherichia* phage vB_EcoM-ep3] | 100% | 0.0 | 99% | [YP_009100020.1](https://www.ncbi.nlm.nih.gov/protein/712913196?report=genbank&log$=prottop&blast_rank=1&RID=BRE9XW7N013) |
| ORF56- | 40688 | 41212 | 174 | 19.03 | putative small terminase subunit | [[](https://blast.ncbi.nlm.nih.gov/Blast.cgi#alnHdr_937533327)*Escherichia* phage vB_EcoM_ECO1230-10] | 100% | 7e-118 | 98% | [YP_009168913.1](https://www.ncbi.nlm.nih.gov/protein/937533310?report=genbank&log$=prottop&blast_rank=1&RID=BREZK1A401N) |

^a^ +, right orientation; -, left orientation.
