# Supplementary material for: Identification and Characterization of Dpo42, a Novel Depolymerase Derived from the Escherichia coli Phage vB_EcoM_ECOO78
Source: Front Microbiol. 2017 Aug 2;8:1460. doi: 10.3389/fmicb.2017.01460 (PMC5539073; doi:10.3389/fmicb.2017.01460)
Supplement: Supplementary file 3 [file Table_3.DOC]

**Table S3. PSI-BLAST iteration of ORF42 in vB_EcoM_ECOO78.**

| No. | Protein description | Length | Score (Bits) | Identities % | Positives% | E |
| --- | --- | --- | --- | --- | --- | --- |
| 1 | EPS-depolymerase OS=*Erwinia* phage phiEa2809 | 939 | 61.6 | 31.0 | 41.0 | 4.0E-6 |
| 2 | Putative EPS depolymerase OS=*Pantoea vagans* (strain C9-1) | 810 | 57.4 | 22.0 | 39.0 | 8.0E-5 |
| 3 | Putative EPS depolymerase OS=*Erwinia* phage vB_EamM_Kwan | 632 | 48.9 | 26.0 | 39.0 | 0.029 |
| 4 | EPS-depolymerase OS=*Erwinia* phage PEp14 | 928 | 48.5 | 28.0 | 43.0 | 0.037 |
