# Supplementary figures and images for: Identification and Characterization of Dpo42, a Novel Depolymerase Derived from the Escherichia coli Phage vB_EcoM_ECOO78
Source: Front Microbiol. 2017 Aug 2;8:1460. doi: 10.3389/fmicb.2017.01460 (PMC5539073; doi:10.3389/fmicb.2017.01460)

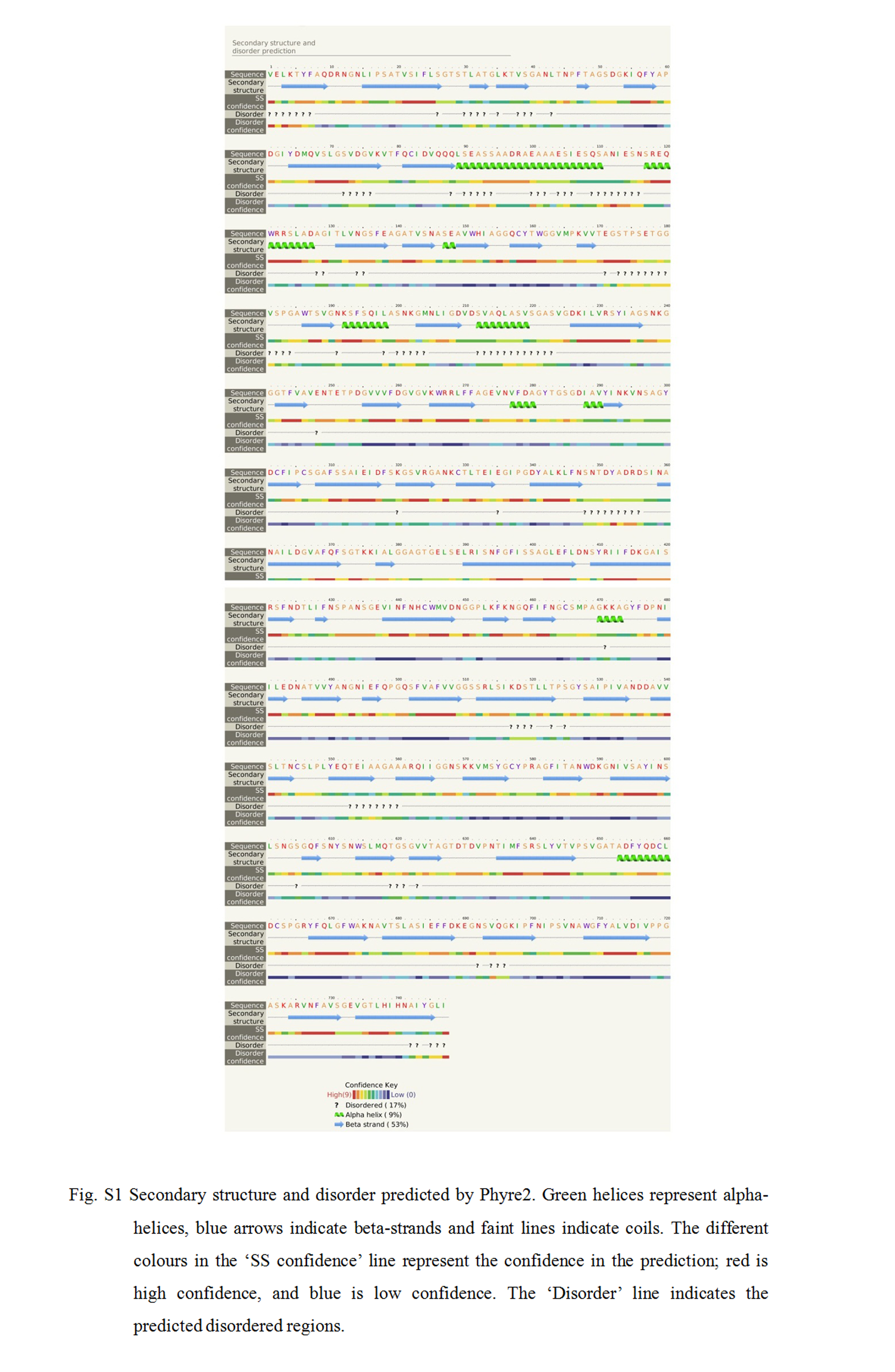

Supplement: Supplementary file 4 [file Image_1.TIF]

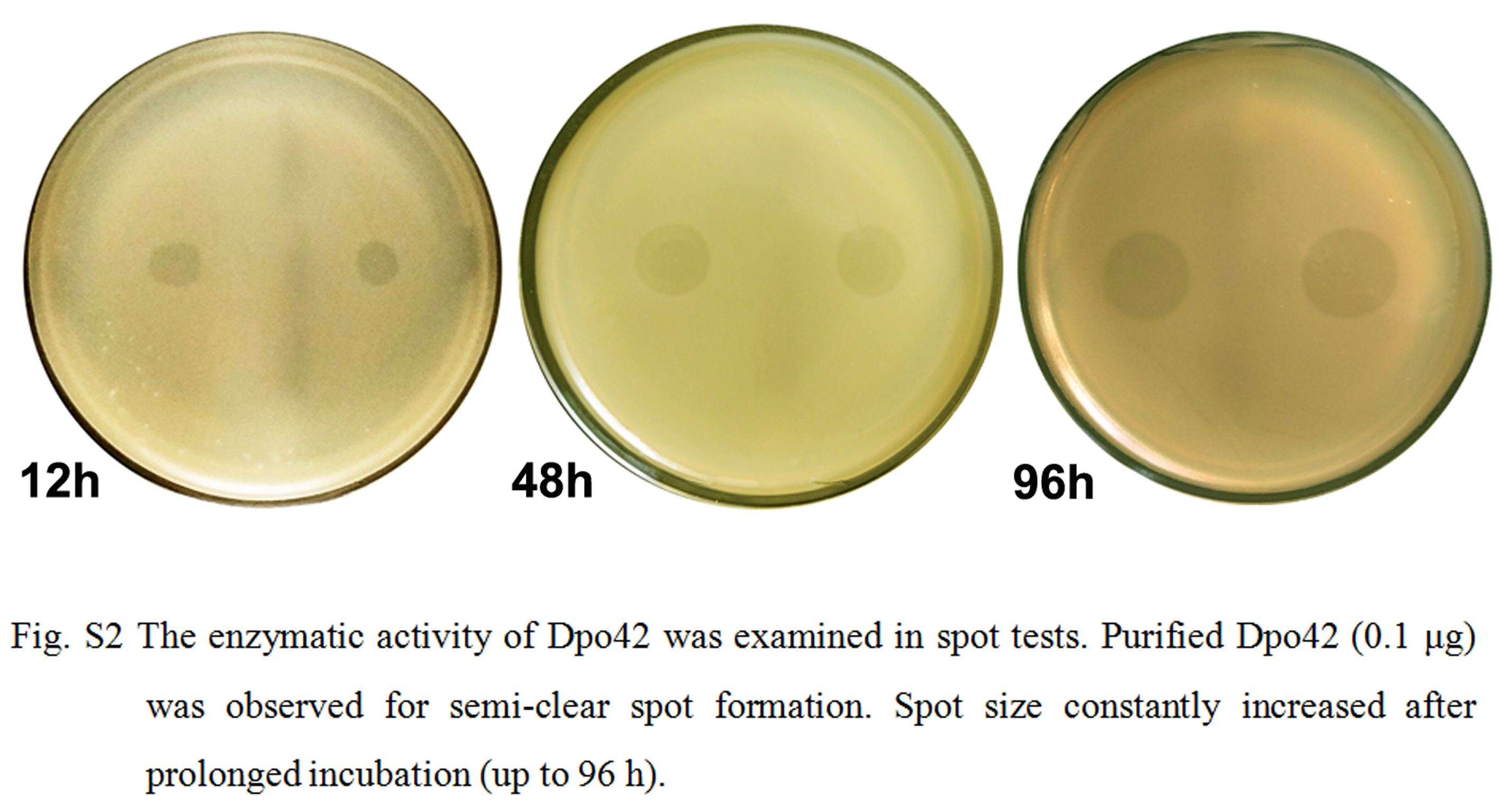

Supplement: Supplementary file 5 [file Image_2.TIF]
